# Millisecond-Delayed Fluorescence in Heavy-Halogen-Substituted TADF Emitters for Air-Pressure Sensing

**DOI:** 10.1021/acsami.6c02198

**Published:** 2026-05-07

**Authors:** Fang Zhao, Christian Hernández-Álvarez, Illia E. Serdiuk, Michał Mońka, Sebastian Mahlik, Marcin Runowski

**Affiliations:** † 467899 Adam Mickiewicz University, Faculty of Chemistry, Uniwersytetu Poznańskiego 8, 61-614 Poznań, Poland; ‡ Institute of Experimental Physics, Faculty of Mathematics, Physics and Informatics, University of Gdansk, Wita Stwosza 57, Gdansk 80308, Poland

**Keywords:** oxygen quenching, optical manometers, halogen substituted organic phosphors, thermally activated delayed fluorescence, charge-transfer states, pressure-dependent photoluminescence

## Abstract

Designing luminescent materials with controllable responses to external stimuli is important for optical sensing applications. Here, two heavy-halogen-substituted donor–acceptor emitters based on a dibenzo­[*a*,*c*]­phenazine-3,6-dicarbonitrile scaffold were synthesized and investigated as low-pressure optical sensors under UV excitation (365 nm). In Zeonex films, both compounds exhibit thermally activated delayed fluorescence (TADF), where the emission intensity strongly depends on oxygen concentration, enabling pressure-dependent luminescence. Reduced pressure suppresses oxygen-induced triplet quenching, resulting in enhanced delayed fluorescence. Halogen substitution modulates the photophysical processes and leads to different sensing performances. The brominated derivative shows a maximum pressure sensitivity of 6.8% mbar^–1^ in the range of 0.1–100 mbar, while the iodinated analogue reaches 1.8% mbar^–1^ between 1 and 700 mbar. The weak temperature dependence allows reliable pressure readout within the investigated temperature range. These results demonstrate that heavy-halogen substitution provides an effective strategy for developing oxygen-regulated TADF materials for optical pressure sensing.

## Graphical abstract

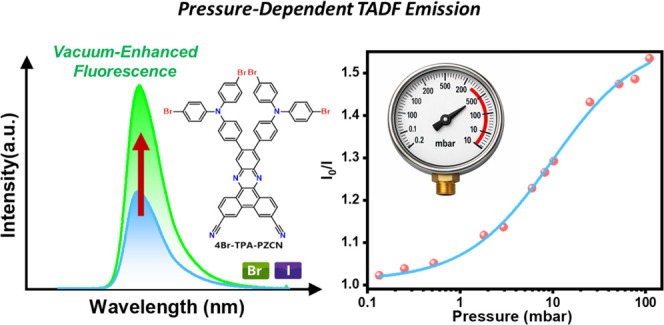

## 1 Introduction

The ability to monitor environmental parameters with high precision and reliability is crucial for a wide range of applications, spanning from medical diagnostics and industrial process control to aerospace engineering.^1–4^ In such contexts, fluctuations in pressure and temperature can directly affect system stability, safety, and performance.^5,6^ With the growing demand for miniaturized and multifunctional devices, optical sensors have gained increasing attention due to their intrinsic advantages, including noncontact operation,^7,8^ immunity to electromagnetic interference,^9^ and ease of integration into compact systems.^10^ Furthermore, signal readouts based on luminescence intensity, lifetime, or spectral ratios enable real-time, high-resolution detection of external stimuli.^11–14^

Over the past decade, the development of new luminescent materials has significantly advanced the performance and versatility of optical sensors. Inorganic materials such as scheelites,^15^ perovskite,^16,17^ and orthovanadates,^18,19^ as well as carbon-based^20,21^ and organic emitters^11,22^ have demonstrated promising photophysical properties. Nevertheless, many of these materials suffer from complex synthesis routes, limited mechanical or chemical stability, or a restricted sensing range. These limitations continue to drive the search for molecular systems that combine synthetic simplicity, high stability, and multifunctional luminescent behavior.

Organic materials whose emission relies on long-lived triplet states (e.g., phosphorescence) are intrinsically sensitive to molecular oxygen.^23^ In its ground state, oxygen exists as a triplet (^3^O_2_), which makes it an efficient quencher of excited triplet states in organic emitters. Diffusion-controlled collisional interactions with oxygen lead to nonradiative deactivation via energy transfer from the excited triplet (*T*_1_) state to ^3^O_2_, thereby quenching phosphorescence and making the luminescence dependent on oxygen concentration and, consequently, on ambient air pressure. Organic photoluminescent materials offer several advantages for optical sensing applications, including structural tunability, low cost, solution processability, and compatibility with flexible substrates. These properties make them attractive candidates for the development of lightweight and versatile optical sensors.^24^ However, in purely organic phosphorescent systems, the radiative decay rate is typically too slow to compete effectively with nonradiative deactivation pathways, apart from quenching by ^3^O_2_.^25^ In addition, the strong susceptibility of organic triplet states to oxygen-induced quenching and other nonradiative processes can significantly reduce emission efficiency.^24^ As a result, the luminescence response to changes in oxygen concentration is weak, limiting sensor sensitivity.

Thermally activated delayed fluorescence (TADF) materials are particularly promising for pressure- and oxygen-sensing applications^26–28^ because their emission is typically faster than phosphorescence but also relies on triplet excitons. In recent years, several TADF systems have been reported to exhibit sensitivity to oxygen concentration or oxygen partial pressure, demonstrating their potential for optical oxygen sensing and pressure-related applications.^29–32^ These studies highlight the capability of TADF emitters to provide efficient optical readouts through oxygen-induced modulation of delayed fluorescence. In TADF systems, the *T*_1_ state formed via intersystem crossing (ISC) is thermally upconverted to *S*_1_ through reverse intersystem crossing (rISC), followed by radiative decay—delayed fluorescence (DF). Interaction with ^3^O_2_ suppresses the DF component by quenching triplet states, providing an optical readout of oxygen pressure. In the presence of oxygen, the key process that is effectively blocked is rISC; therefore, by adjusting the rISC rate to the oxygen diffusion in a given medium, one can control both the sensitivity and the operational pressure range of a TADF-based sensor.

An ideal TADF-based pressure sensor must satisfy several key criteria. First, a long DF lifetime (τ_DF_) is required to maximize the probability of interaction with ^3^O_2_, whose diffusion time varies widely across media, from microseconds to seconds. This can be achieved by carefully tuning the *S*_1_–*T*_1_ energy gap (Δ*E*_ST_) of a TADF material. While a small Δ*E*_ST_ < 0.2 eV is necessary to enable rISC, it should not be excessively small (>0.1 eV), as this may shorten τ_DF_. Therefore, a moderately small Δ*E*_ST_ in the range of 0.1–0.2 eV is expected to provide τ_DF_ in a millisecond range. Second, the prompt fluorescence (PF) quantum yield should be suppressed to extend the emission lifetime further and enhance oxygen sensitivity. Another, third crucial criterion is maximizing the *T*_1_ yield via dominating ISC. In most molecular sensors studied before, this is achieved using precious heavy metals like Pt or Pd.^33^ Alternatively, in organic TADF sensors, ISC can be enhanced using abundant heavy halogen atoms. Halogenation–offers a powerful strategy to modulate spin–orbit coupling, charge-transfer efficiency, and intermolecular interactions.^34^ The heavy-atom effect introduced by bromine or iodine enhances ISC and rISC, through increased spin–orbit coupling and thereby regulating emission wavelength, lifetime, and environmental sensitivity.^35–37^

In this work, the first and second criteria are achieved by employing a donor–acceptor (D–A) architecture forming a red TADF emitter. We use a TPA-PZCN scaffold^38^ featuring a strong dibenzo­[*a*,*c*]­phenazine-3,6-dicarbonitrile (PZCN) acceptor with two relatively weak but sterically hindered triphenylamine (TPA) donors. TPA is only partially conjugated with the acceptor, which limits HOMO–LUMO overlap, providing (1) Δ*E*_ST_ of 0.13–0.14 eV and (2) reduced *S*_1_–*S*_0_ oscillator strength, i.e., low fluorescence rate. To adjust this molecule for pressure sensing, we incorporate either two bromine (compound 4Br-TPA-PZCN, Scheme 1) or two iodine atoms (4I-TPA-PZCN) into both TPA units to (3) increase the triplet population, DF lifetime and thus sensitivity to O_2_. The compounds were examined under variable pressure and temperature conditions using 365 nm excitation, and quantitative correlations between luminescence intensity ratios (LIR) and stimuli were established. These investigations examine the pressure-dependent luminescence behavior of heavy-halogen-substituted TADF emitters and consider the effect of temperature on their emission, in the context of optical pressure sensing.

**Scheme 1:**
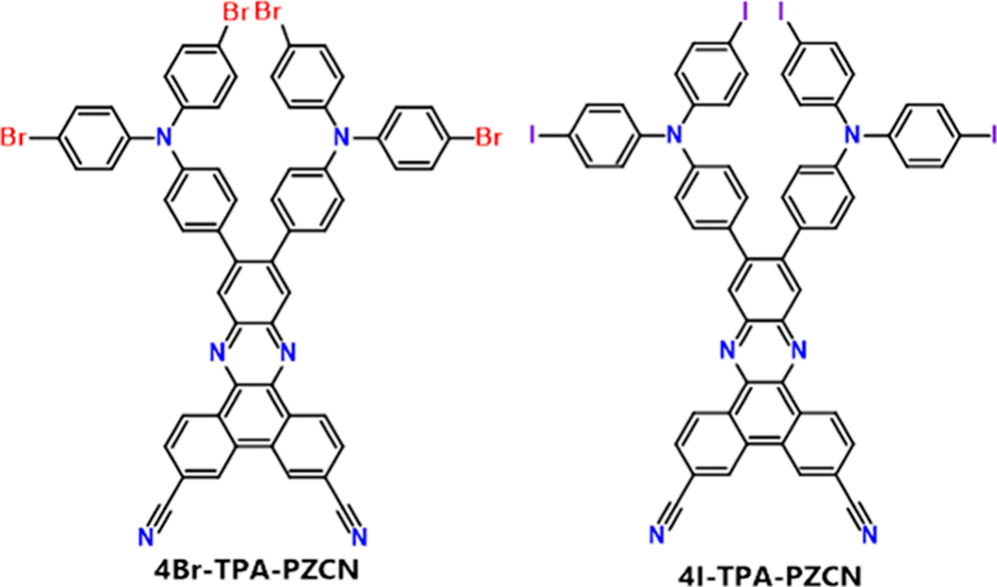
Canonic Structures of Studied TADF Molecules

## 2 Experimental Section

### 2.1 Materials and Synthesis

Materials. Zeonex (ZNX, Zeonex480R, density = 1.01 g/cm^3^) was purchased from Zeon Europe GmbH. Reagents for synthesis were of corresponding grade and were used as supplied.

Synthesis of emitters. 4Br-TPA-PZCN and 4I-TPA-PZCN were prepared in a single step via halogenation of TPA-PZCN synthesized according to a previously reported protocol,^38^ the reaction scheme is shown in Figure S1, Supporting Information.

#### 2.1.1 Halogenation Procedure

The mixture of TPA-PZCN (82 mg, 0.1 mmol), *N*-bromosuccinimide (79 mg, 0.44 mmol, for 4Br-TPA-PZCN) or *N*-iodosuccinimide (100 mg, 0.44 mmol, for 4I-TPA-PZCN) in 0.8 mL of chloroform was placed in a pressure vessel (*V* = 5 mL), closed, and stirred at 80 °C for 12 h in dark. Solvent was evaporated and the residue was treated with methanol. The resulting precipitate was dried and purified by recrystallization from CHCl_3_–MeOH mixture or by the column chromatography using appropriate hexane-chloroform mixtures as eluents.

#### 2.1.2 11,12-bis­(4-(di­(4-bromophenyl)­amino)­phenyl)­dibenzo­[*a*,*c*]­phenazine-3,6-dicarbonitrile (4Br-TPA-PZCN)

Orange crystalline powder, recrystallized from CHCl_3_–MeOH, yield 93%. ^1^H NMR (500 MHz, CDCl_3_, δ): 6.98–7.08 (m, 12H), 7.21–7.27 (m, 4H), 7.38–7.44 (m, 8H), 8.07 (d, 2H, *J* = 8.2 Hz), 8.42 (s, 2H), 8.87 (s, 2H), 9.57 (d, 2H, *J* = 8.2 Hz) (Figure S5, Supporting Information). ^13^C NMR spectrum unavailable due to low solubility. MALDI-TOF MS *m*/*z*: calcd for C_58_H_32_N_6_Br_4_, 1128.95 [M + H]^+^; found, 1128.97. Elem. Anal. Calcd, %: C 61.51; H 2.85; N 7.42. Found, %: C 61.42; H 2.89; N 7.37.

#### 2.1.3 11,12-bis­(4-(di­(4-iodophenyl)­amino)­phenyl)­dibenzo­[*a*,*c*]­phenazine-3,6-dicarbonitrile (4I-TPA-PZCN)

Red crystalline powder, purified by column chromatography with the CHCl_3_-hexane eluent, yield 86%. ^1^H NMR (500 MHz, CDCl_3_, δ): 6.85–6.93 (m, 4H), 7.03–7.09 (m, 4H), 7.10–7.17 (m, 4H), 7.20–7.26 (m, 4H), 7.30–7.36 (m, 4H), 7.54–7.61 (m, 4H), 8.03 (d, 2H, *J* = 7.9 Hz), 8.39 (s, 2H), 8.81 (s, 2H), 9.52 (d, 2H, *J* = 7.9 Hz) (Figure S6, Supporting Information). ^13^C NMR (125 MHz, CDCl_3_, δ): 147.33, 146.98, 146.88, 142.28, 138.49, 138.30, 134.26, 133.84, 131.06, 130.95, 130.38, 129.84, 129.61, 127.54, 127.31, 126.16, 125.96, 124.97, 123.96, 123.04, 118.45, 114.05 (Figure S7, Supporting Information). MALDI-TOF MS *m*/*z*: calcd for C_58_H_32_N_6_I_4_, 1320.89 [M + H]^+^; found, 1320.91. Elem. Anal. Calcd, %: C 52.75; H 2.44; N 6.36. Found, %: C 52.81; H 2.52; N 6.28.

#### 2.1.4 Preparation of Polymer Films

The thin films were prepared from a solution of ZEONEX in toluene mixed with a stock solution containing the emitter. The concentration of ZNX was 200 mg mL^–1^, while the emitter concentration was 1 mg L^–1^. The solutions were combined to obtain a final emitter loading of 0.1 wt % relative to the solid polymer matrix. The resulting mixture was drop-cast onto a glass substrate and left to dry for 20 h at 50 °C, yielding the final film suitable for measurements.

### 2.2 Characterization

#### 2.2.1 Physicochemical Analysis

The ^1^H and ^13^C NMR spectra were recorded on a Bruker AVANCE III 500 (500 MHz) instrument using CDCl_3_ as the solvent with Me4Si as the internal standard. MALDITOF mass spectrum was obtained using a Bruker Autoflex MaX mass spectrometer with 2,5-dihydroxy-benzoic acid (DHB) matrix. Elemental analysis was performed on an Elementar Vario El Cube CHNS analyzer. For NMR and mass spectra, see Supporting Information.

#### 2.2.2 Sample Preparation

4Br-TPA-PZCN and 4I-TPA-PZCN were dispersed in ZNX films via drop-casting. The emitters were first dissolved in chloroform under heating and sonication to obtain clear solutions. An appropriate amount of each solution was then added to a toluene solution of ZNX and carefully mixed to avoid bubble formation, yielding a final emitter concentration of 0.1 wt % relative to the dry polymer. The resulting solution was drop-cast onto a 2 × 2 cm microscope glass slide and allowed to dry slowly under ambient conditions for 10 h to ensure slow solvent evaporation. Subsequently, the film was transferred to a hot plate and annealed at 50 °C overnight. After drying, the film was carefully peeled from the glass substrate.

#### 2.2.3 Photophysical Characterization

Steady-state photoluminescence (PL) spectra and absolute PL quantum yields were recorded using an FS5 spectrofluorometer (Edinburgh Instruments) equipped with an integrating sphere. Time-resolved PL measurements were performed using a customized system^39^ consisting of a pulsed YAG:Nd laser (PL2251A, EKSPLA) coupled with an optical parametric generator (PG 401/SH) as an excitation light source and 2501S grating spectrometer (Bruker Optics) combined with the streak camera system (C4334-01 Hamamatsu) as a detection unit. The system was equipped with a double-stage high-vacuum pump (Edwards T-Station 85) and a helium cryostat for low-temperature measurements. Rate constants for photophysical processes were calculated using previously described equations.^40,41^

#### 2.2.4 Pressure Tests

The sample was excited using a standard 450 W xenon lamp, combined with a monochromator, to select an excitation wavelength of 365 nm. Emission spectra were recorded in the visible range using an Andor Shamrock 500 spectrometer coupled to an Andor Newton silicon CCD camera. For pressure-dependent measurements, the sample was placed in a vacuum chamber connected to a HiCube 300 H Neo (Pfeiffer Vacuum) vacuum pump, equipped with CCT361 (Pfeiffer Vacuum) and CCT364 (Pfeiffer Vacuum) pressure sensors.

#### 2.2.5 Temperature Tests

Temperature-dependent PL spectra were recorded using the same optical setup as for pressure experiment. The sample was excited at 365 nm, and the emission was collected using the CCD camera. For the temperature-dependent measurements at ambient pressure the sample was placed in a heating–cooling stage (Linkam THMS600). Whereas for the temperature-dependent measurements under vacuum conditions, the sample was placed in a vacuum chamber of the cryostat (the same one as for the pressure tests), where the temperature was adjusted in a vacuum-type nitrogen cryostat LN1200 (from Advanced Research Systems).

## 3 Results and Discussion

### 3.1 Photoluminescence and TADF

For spectral feature investigations and sensing tests, the emitters were dispersed in ZEONEX (ZNX) films at a 0.1 wt % concentration. ZNX provides an optically transparent and mechanically robust polymeric matrix with good oxygen permeability. In such a medium, both emitters show broad-band charge-transfer (CT) type red emission with photoluminescence (PL) maxima at λ_em_ = 554 nm (4Br-TPA-PZCN) and 545 nm (4I-TPA-PZCN) (Figure 1a, Table 1). The excitation profiles exhibit broad bands, demonstrating that the materials can be efficiently excited over a wide UV–vis spectral range below 550 nm. A key signature of TADF, namely the same spectral shape of PF and DF, is clearly observed in the time-resolved emission spectra: for both compounds, the spectra recorded within the first 50 ns (PF) and 1–10 ms (DF) are identical (Figures 1b,c and S2b,c, Supporting Indormation), confirming that the long-lived component is delayed fluorescence rather than phosphorescence. Additional proof of the thermally activated nature of DF is provided by low-temperature experiments: when cooled down to 10 K, DF disappears completely and a strongly red-shifted phosphorescence is observed (Figure S3, Supporting Information). These findings also argue against triplet–triplet annihilation (TTA), since TTA-based DF can still occur under cryogenic conditions.

**Figure 1:**
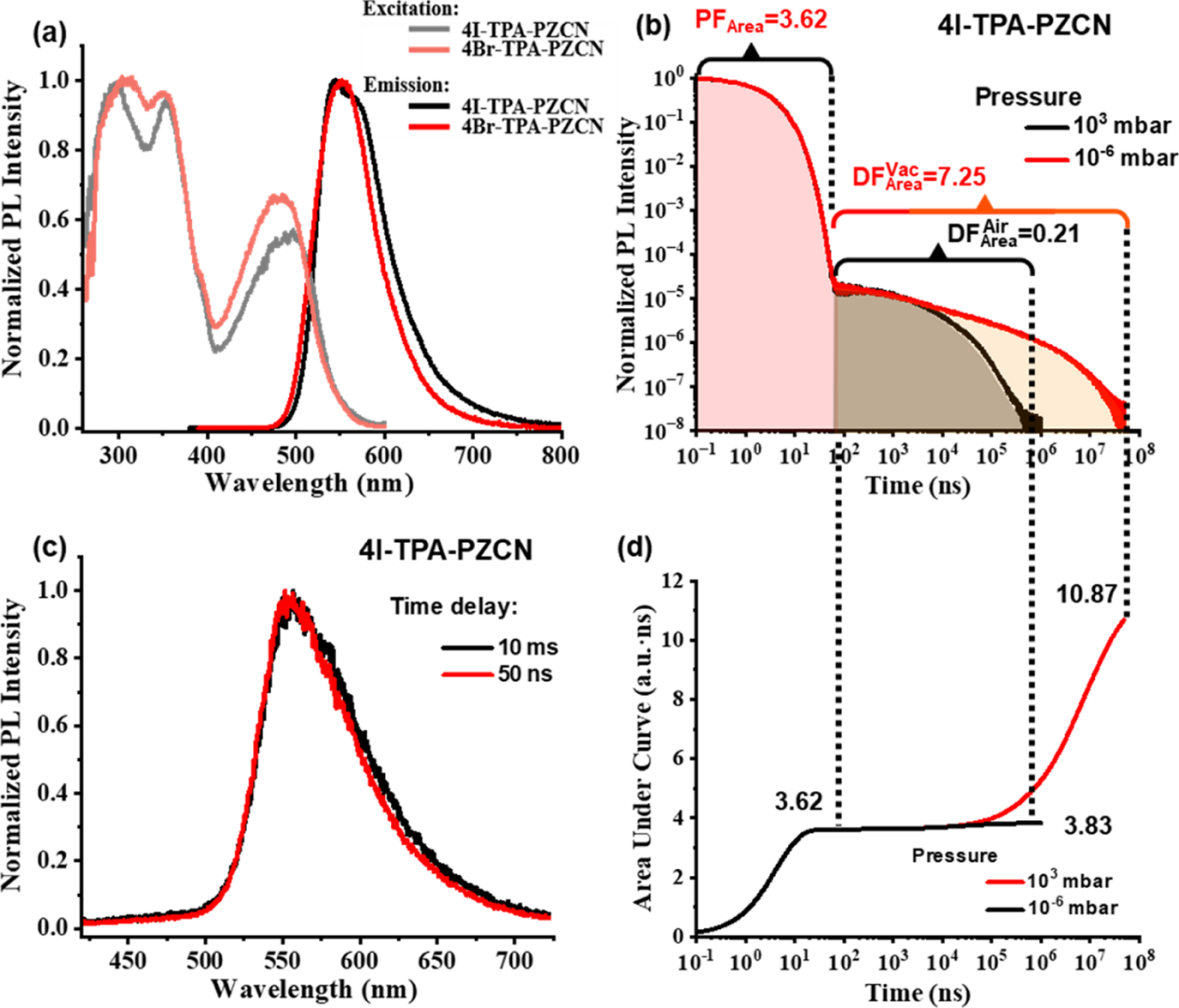
Photoluminescence features of studied emitters in ZNX films (*c* = 0.1% w/w). (a) Steady-state excitation (λ_obs_ = 610 nm) and emission (λ_exc_ = 330 nm) spectra. (b) Photoluminescence intensity decays of 4I-TPA-PZCN (λ_exc_ = 330 nm, 298 K) at normal and reduced pressure. (c) Time-resolved emission spectra of 4I-TPA-PZCN (λ_exc_ = 330 nm, 298 K) recorded with different delays. (d) Integrated emission intensities calculated from the areas under PL decay curves of 4I-TPA-PZCN at normal and reduced pressure.

**Table 1:** Photoluminescent Parameters under Ambient and Reduced Pressure^a^

			PLQY										
	*p* [bar]	λ_em_ [nm]	total [%]	PF [%]	DF [%]	DF/PF	τ_PF_ [ns]	τ_DF_ [ms]	*k*_r_ [10^7^ s^–1^]	*k*_ISC_ [10^7^ s^–1^]	φ_ISC_ [%]	*k*_rISC_ [10^2^ s^–1^]	φ_rISC_ [%]
4Br-TPA-PZCN	10^–9^	554	99	49.6	49.4	0.99	5.01	7.1	9.8	9.8	49	2.9	100
	1.01	554	52	49.6	2.2	0.04	5.01	0.018	9.8				4.4^a^
4I-TPA-PZCN	10^–9^	545	70	22.7	47.3	2.1	3.45	10.2	6.6	20.1	69	2.5	100
	1.01	545	24	22.7	1.3	0.06	3.45	0.019	6.6				2.7^a^

a Calculated using the area under DF decay curves as *S*_air_/*S*_vac._*100%.

Table 1 summarizes the spectral and photophysical parameters of emitters under the reduced pressure (10^–9^ bar). PLQY values reach almost 100% for 4Br-TPA-PZCN and 70% for 4I-TPA-PZCN. The PF lifetimes are in the few-nanosecond range, with τ_PF_ = 5.0 ns (Br) and 3.45 ns (I). The reduction in τ_PF_ upon substitution of Br with I evidence the significant role of the heavy-atom effect in the *S*_1_ deactivation dynamics. Specifically, this leads to a 2-fold increase in the ISC rate constant, from *k*_ISC_ = 9.8 × 10^7^ s^–1^ to 20 × 10^7^ s^–1^. At the same time, the radiative decay rate (*k*_r_) decreases by more than 20% (Table 1). These changes demonstrate the more substantial heavy-atom effect of iodine compared to bromine: (1) the PF quantum yield is reduced by more than 2-fold, (2) triplet states are generated at twice the rate, and (3) the intersystem crossing quantum yield (φ_ISC_) increases from 49% to 69% (Table 1). Consequently, 4I-TPA-PZCN more effectively satisfies the above-mentioned criteria 2 and 3 for pressure sensing.

As for the delayed fluorescence, it occurs on the millisecond time scale, and its quantum yield remains comparable for both compounds at approximately 47%. Upon substitution of Br with I, the DF lifetime increases from τ_DF_ = 7.1 to 10.2 ms. This prolongation originates from the above-mentioned higher φ_ISC_ in 4I-TPA-PZCN, while the rate constant of the main deactivation route of *T*_1_, namely rISC, is even slightly reduced: *k*_rISC_ = 290 s^–1^ and 250 s^–1^, respectively. These factors cause the iodinated emitter to possess a longer DF, as defined by the first criterion.

It should be noted that the uniqueness of these TADF materials lies in the unusual balance of excited-state rate constants: the ISC rate is relatively high, while the rISC rate is extremely low, yet still effectively competes with nonradiative deactivation of the excited states. As a result, the ratio of ISC to rISC rate constants is huge, exceeding 3 × 10^5^ for 4Br-TPA-PZCN and 8 × 10^5^ for 4I-TPA-PZCN. This implies that for each *S*_1_ state recovered from *T*_1_ via rISC, hundreds of thousands of new triplet states are generated through ISC, providing both extended interaction time and a significantly increased population of excited states available for quenching by molecular oxygen.

On the molecular level, such properties can be explained by the combination of large *S*_1_–*T*_1_ energy gap (Δ*E*_ST_) and heavy-atom effect. In fact, the PL measurements at 10 K (Figure S3, Supporting Information) reveal that the Δ*E*_ST_ values reach 0.29 eV (4Br-TPA-PZCN) and 0.27 eV (4I-TPA-PZCN). Such values are significantly larger than those usually considered as upper limit for efficient TADF in light-atom organic emitters (0.20 eV). The observation of TADF despite these large Δ*E*_ST_ values confirms and emphasized the key role of the heavy-atom effect. The presence of multiple heavy atoms enhances strongly spin–orbit coupling (SOC), which has main effect on ISC but also facilitates rISC and enables TADF even when Δ*E*_ST_ is so large.

As a result, when measurements are performed under normal air pressure, the photophysical behavior changes. The PLQY values in air decrease substantially compared to vacuum, reaching 52% for 4Br-TPA-PZCN and 24% for 4I-TPA-PZCN. The DF component is strongly suppressed and its lifetime decreases to the microsecond time scale to τ_DF_ ≈ 180–190 μs (Figures 1b and S2b, Supporting Information). The rISC quantum yield (φ_rISC_) is reduced to only a few percent (Table 1), indicating that quenching by ^3^O_2_ becomes the dominant deactivation pathway of *T*_1_.

Finally, the PLQY and integrated decay-area ratios under vacuum and air conditions quantitatively indicate the pressure sensitivity of the emitters. Under the removal of oxygen, the total emission enhancement factor (vacuum/air) reaches 1.8 for 4Br-TPA-PZCN and 2.8 for 4I-TPA-PZCN (Figures 1d, S2d, and Table S1, Supporting Information). Such findings support the expectation that the iodinated emitter will exhibit a stronger response to changes in oxygen partial pressure. On this basis, the following section focuses on the quantitative evaluation of optical air-pressure sensing performance under controlled pressure conditions.

Note that estimation of the ISC and rISC rate constants and ISC quantum yield, which is done using DF to estimate the amount of *T*_1_ states formed, is not feasible under air conditions (ambient pressure) in this case, as the triplet population is strongly perturbed by oxygen quenching in the system studied. Hence, the corresponding values are not given in a Table 1.

### 3.2 Optical Air-Pressure Sensing

The investigated materials exhibit oxygen-induced fluorescence quenching, a phenomenon common in some organic molecules. As a result, their fluorescence intensity increases in vacuum and decreases with increasing oxygen concentration (pressure). This effect arises because molecular oxygen can accept energy from excited luminophores, intercepting the excitation energy before radiative emission occurs (Figure 2).^42,43^

**Figure 2:**
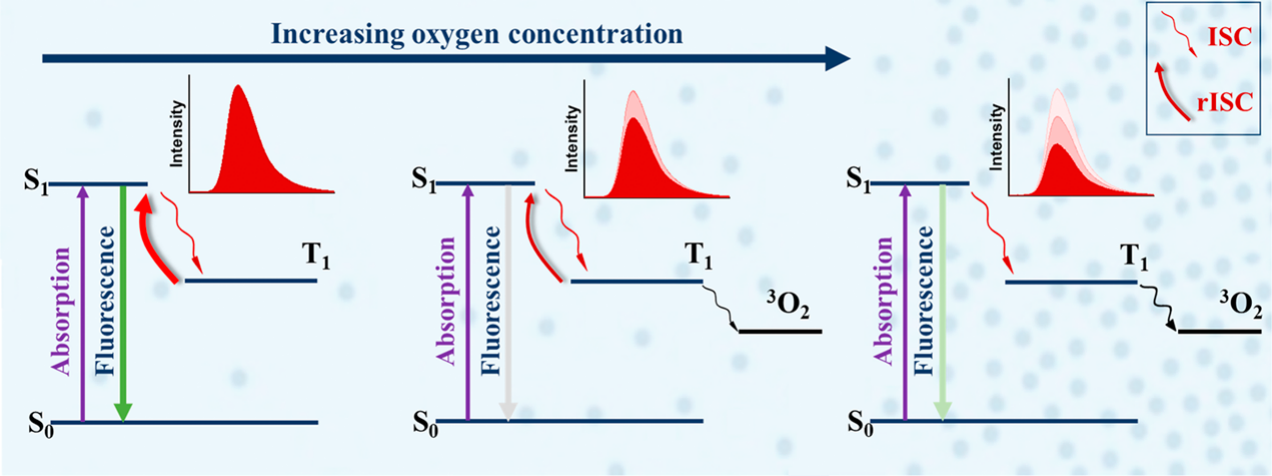
Mechanism of oxygen-dependent fluorescence quenching under variable pressure.

Hence, to evaluate the potential of the synthesized 4Br-TPA-PZCN and 4I-TPA-PZCN organic phosphors as optical low-pressure (vacuum) sensors, their pressure-dependent emission behaviors were systematically investigated. As shown in Figure 3a,c, both samples exhibit a gradual decrease in their normalized luminescence intensity with increasing pressure, reflecting the higher oxygen concentration at higher pressures. This oxygen-induced quenching is more pronounced for 4I-TPA-PZCN, suggesting stronger susceptibility to oxygen-mediated nonradiative decay. To quantify the optical response, the LIR technique was as a reliable calibration method.^44,45^ Due to the presence of only a single emission band, the LIR parameter was defined as LIR = *I*_0_/*I*, where *I*_0_ corresponds to the emission intensity measured under vacuum (i.e., the lowest pressure), and *I* is the intensity at a given pressure. As shown in Figure 3b, for the 4Br-TPA-PZCN, the LIR remains nearly constant at low pressures, followed by a rapid increase and then gradual saturation; for the 4I-TPA-PZCN, the LIR also remains stable at low pressures, then increases sharply (Figure 3d). To further compare the pressure-response behaviors of the two samples, the empirical fitting model

1 $$LIR=A+\frac{B}{1-{\left(p/p_{0}\right)}^{n}}$$

where *A* and *B* are fitting constants related to the baseline and pressure-dependent response amplitude, respectively; *p* denotes the applied pressure, *p*_0_ represents the characteristic pressure of the system, and *n* is a dimensionless exponent describing the pressure-response nonlinearity. The corresponding fitting parameters are summarized in Table S2. The fits are excellent, and the selected pressure intervals (4Br-TPA-PZCN: 0.1–100 mbar; 4I-TPA-PZCN: 1–700 mbar) ensure that all measurements fall within the physically meaningful regime of the model. These ranges were chosen to capture the full dynamic response of each material while avoiding pressures at which the LIR either remains fully saturated or exhibits negligible variation.^45,46^ For 4Br-TPA-PZCN, pressures below 0.1 mbar would show little additional change due to the near-zero oxygen concentration, whereas pressures above 100 mbar begin to approach the quenching saturation limit. Similarly, for 4I-TPA-PZCN, pressures below 1 mbar fall below the sensitive response window, and pressures above 700 mbar approach saturation, where the LIR variation slows considerably.

**Figure 3:**
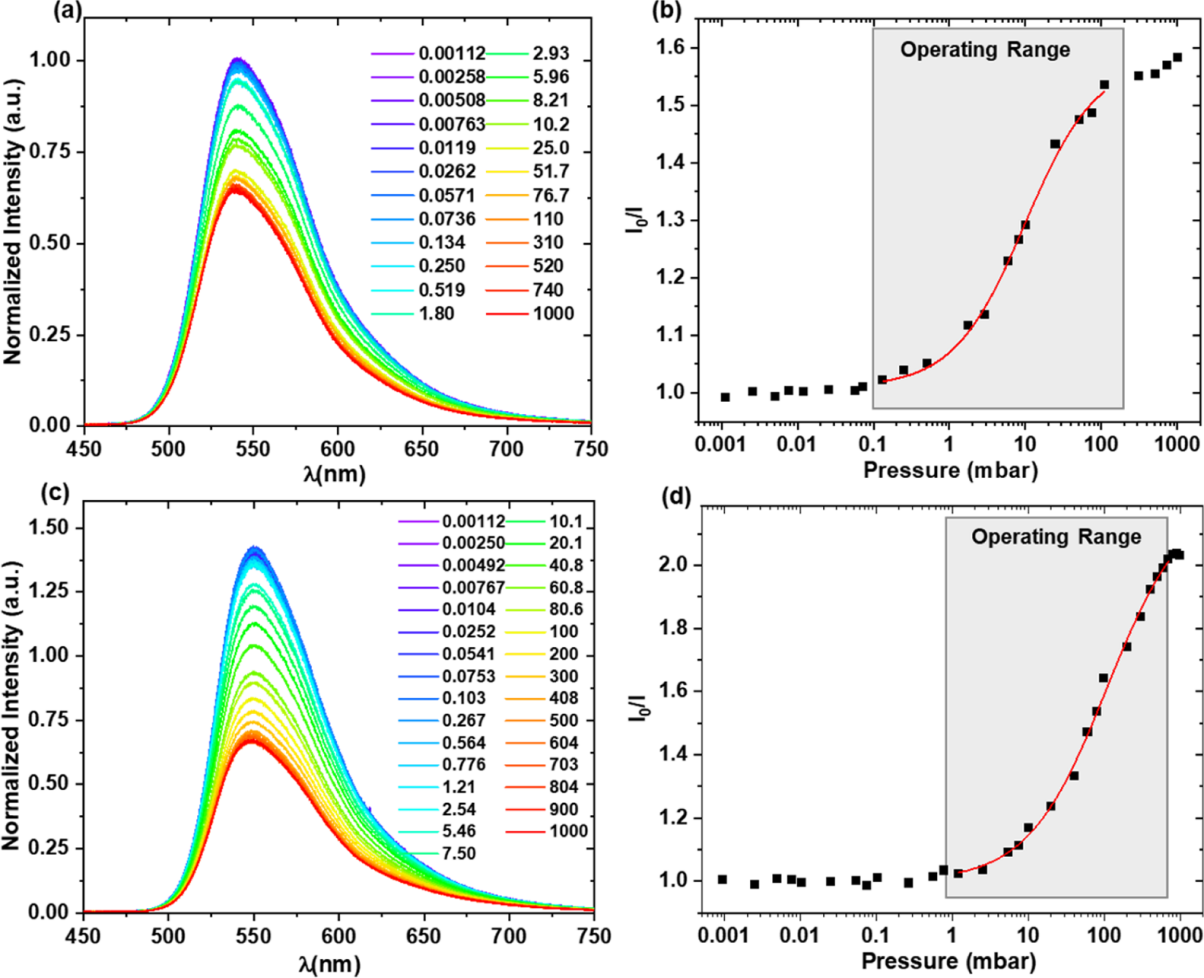
Emission spectra of the synthesized organic compounds, i.e. 4Br-TPA-PZCN (a) and 4I-TPA-PZCN (c), recorded in the visible range under 365 nm excitation; LIR profiles as a function of pressure (b,d) for the 4Br-TPA-PZCN (b) and 4I-TPA-PZCN (d) derivatives.

A comparison of the fitting parameters reveals distinct response characteristics. 4Br-TPA-PZCN features a smaller characteristic pressure *p*_0_, indicating a strong response at low pressures, together with larger values of |*B*| and *n*, which result in a steeper LIR rise. In contrast, the 4I-TPA-PZCN displays a higher characteristic pressure and a more gradual LIR increase, consistent with its higher operating-pressure bias.

To evaluate the air-pressure sensing capability and performance of the developed optical sensor, its pressure sensitivities were determined. The following equations were used to calculate both the absolute and relative pressure sensitivities (*S*_a_ and *S*_r_)^44,47,48^

2 $$S_{a}\left(p\right)=\frac{dLIR}{dp}$$

3 $$S_{r}\left(p\right)=100\%\times \frac{1}{LIR}\frac{dLIR}{dp}$$

Figure 4a,b illustrate the *S*_a_ and *S*_r_ as functions of pressure for both samples. The maximum *S*_r_ approaches 6.8% mbar^–1^ for the 4Br-TPA-PZCN and 1.8% mbar^–1^ for the 4I-TPA-PZCN in the minimum-pressure range, indicating strong responsiveness in that interval. And the pressure dependence of *S*_a_ and *S*_r_ reflects the underlying quenching mechanism. At low pressures, the low oxygen concentration makes the luminescence highly responsive to small pressure variations, leading to the maximum *S*_r_ observed for both samples in this region. As pressure increases, oxygen quenching gradually approaches saturation, leading to a systematic decrease in both *S*_a_ and *S*_r_.

**Figure 4:**
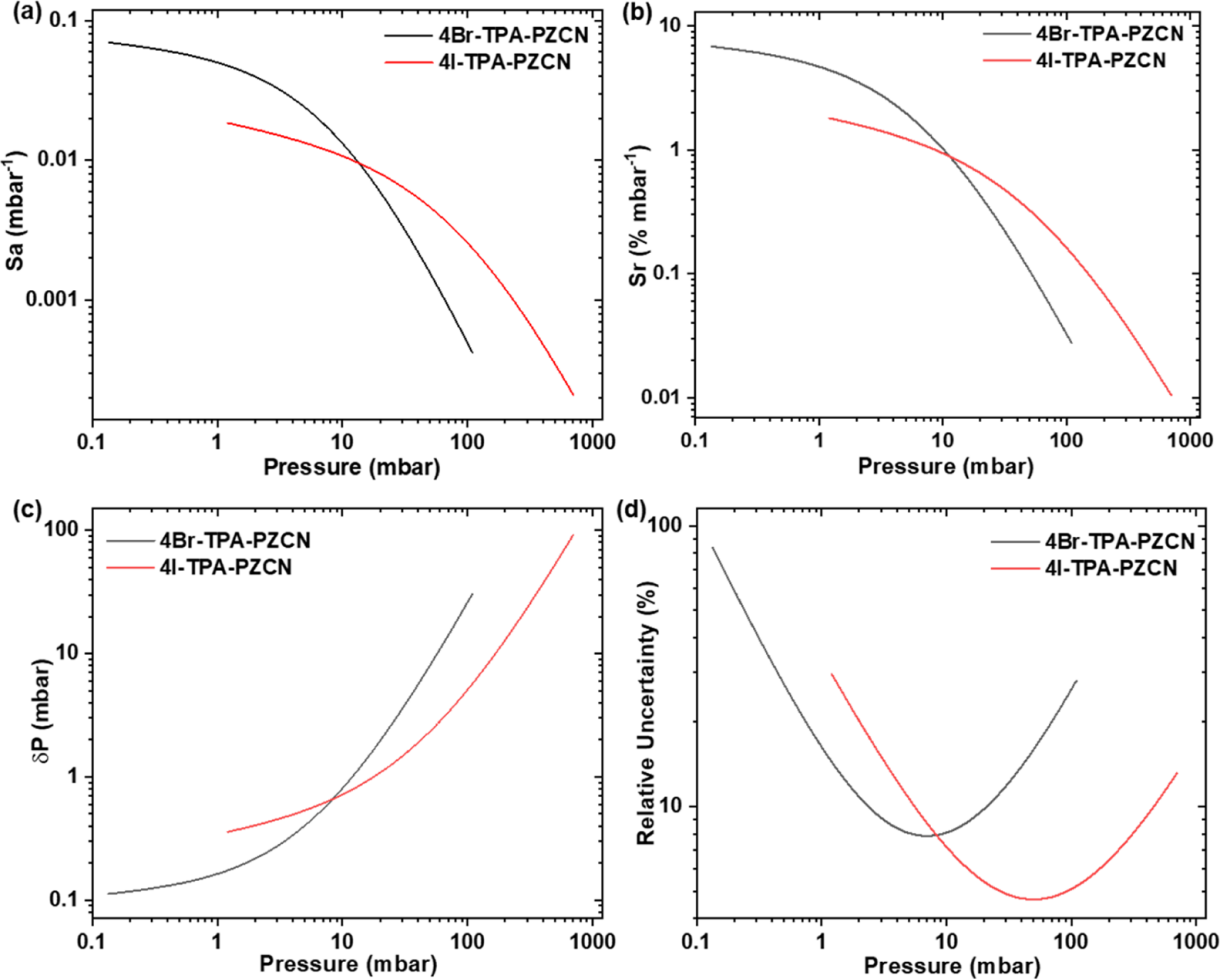
Absolute (a) and relative (b) sensitivity for the 4Br-TPA-PZCN and 4I-TPA-PZCN compounds as a function of pressure. Absolute uncertainty-sensing resolution (c) and relative uncertainty in % (d) for both materials, plotted as a function of pressure.

These results demonstrate that 4Br-TPA-PZCN is highly effective at detecting small fluctuations in near-vacuum pressure, whereas 4I-TPA-PZCN operates over a higher-pressure regime. This contrasting behavior reflects the influence of halogen substitution on the balance between triplet quenching and reverse intersystem crossing, and highlights the importance of selecting emitters according to the target pressure range when designing oxygen-regulated TADF-based optical pressure sensors.

To assess practical sensing performance, the pressure-detection resolution δ*p* was estimated using established formulas^44,48–50^

4 $$\delta p=\frac{1}{S_{r}}\frac{\delta LIR}{LIR}$$

5 $$\frac{\delta LIR}{LIR}=\sqrt{{\left(\frac{{\delta I}_{0}}{I_{0}}\right)}^{2}+{\left(\frac{\delta I}{I}\right)}^{2}}$$

Figure 4c,d show the absolute uncertainty δ*p* and relative uncertainty for both samples. The 4Br-TPA-PZCN achieves its minimum relative uncertainty of 7.86% at 6 mbar, whereas the 4I-TPA-PZCN reaches 4.67% at 50 mbar. These minimal values correspond well with the pressure ranges where each material shows the most pronounced *S*_r_, demonstrating consistency between the sensitivity analysis and the uncertainty evaluation.

As for the response time of the proposed pressure sensors, it is limited by the longest component of the emission decay, corresponding to the delayed fluorescence lifetime. According to the Figure 1, under vacuum conditions, this component reaches approximately 30–40 ms. With increasing air pressure, the delayed fluorescence is progressively quenched due to oxygen-induced triplet quenching, leading to a significant shortening of the response time to approximately 300 μs at ambient pressure.

### 3.3 Influence of Temperature on the Pressure Sensor Parameters

The temperature-dependent luminescence response originates from thermal modulation of the excited-state dynamics of luminophores. With increasing temperature, nonradiative relaxation pathways-such as vibrational and rotational motions-generally become more efficient, which may lead to a gradual reduction in fluorescence intensity.^51^ However, the extent of this effect strongly depends on the molecular structure and excited-state landscape of a given system and does not necessarily involve pronounced spectral changes. In donor–acceptor luminophores, temperature variations can influence the balance between radiative and nonradiative decay channels by modulating molecular motions, intermolecular interactions, and the accessibility of thermally activated excited states. Depending on the rigidity of the molecular framework and the surrounding environment, these effects can range from negligible to moderate, resulting in diverse temperature-dependent luminescence behaviors.

Temperature-dependent photoluminescence measurements were performed on 4Br-TPA-PZCN and 4I-TPA-PZCN at ambient pressure to quantitatively evaluate their thermal response. In addition, complementary temperature-dependent measurements were carried out under vacuum conditions, i.e. at pressure of ≈10^–6^ bar (Figure S8, Supporting Information), to further assess the intrinsic thermal behavior in the absence of oxygen. As shown in Figure 5a,c, both materials exhibit nearly invariant emission spectra over the investigated temperature range, with only a slight attenuation of the overall emission intensity at elevated temperatures. It indicates that thermal effects introduce only marginal modulation of the excited-state deactivation pathways under these conditions. Whereas in the case of temperature-dependent measurements under vacuum conditions, we did not detect any decrease of luminescence intensity (see Figure S8). The comparison with vacuum measurements reveals that this slight intensity decrease observed in air originates predominantly from enhanced oxygen diffusion at elevated temperatures, which increases the quenching rate of triplet states and reduces the delayed fluorescence contribution. Compared with 4Br-TPA-PZCN, 4I-TPA-PZCN displays a slightly more pronounced intensity attenuation, which can be explained by enhanced nonradiative decay arising from larger-amplitude *C*–*I* vibrational modes at increased temperatures. To quantify the thermal response, the LIR method was employed using the ratio *I*_0_/*I*, where *I*_0_ corresponds to the emission intensity measured at room temperature (used as the reference condition), and *I* is the intensity at a given temperature. As seen in Figure 5b,d, both compounds exhibit monotonic but shallow LIR-temperature dependences across the entire temperature range. The corresponding LIR-T relationships can be empirically described by a second-order polynomial function^52^

6 $$LIR=A_{0}-A_{1}\cdot T+A_{2}\cdot T^{2}$$

with all fitting parameters provided in Table S2 (Supporting Information). The excellent agreement between experimental data and the quadratic fits confirms the absence of abrupt thermal transitions, while the small fitting coefficients indicate a limited sensitivity of LIR to temperature variations. Note that due to the absence of an appropriate physical model to conform the changes of the derived optical sensing parameters used (i.e., LIR), we simply used the empirical exponential and polynomial functions to correlate them with pressure and temperature, as commonly applied in different reports dealing with luminescence manometry and thermometry.^44,48,53^

**Figure 5:**
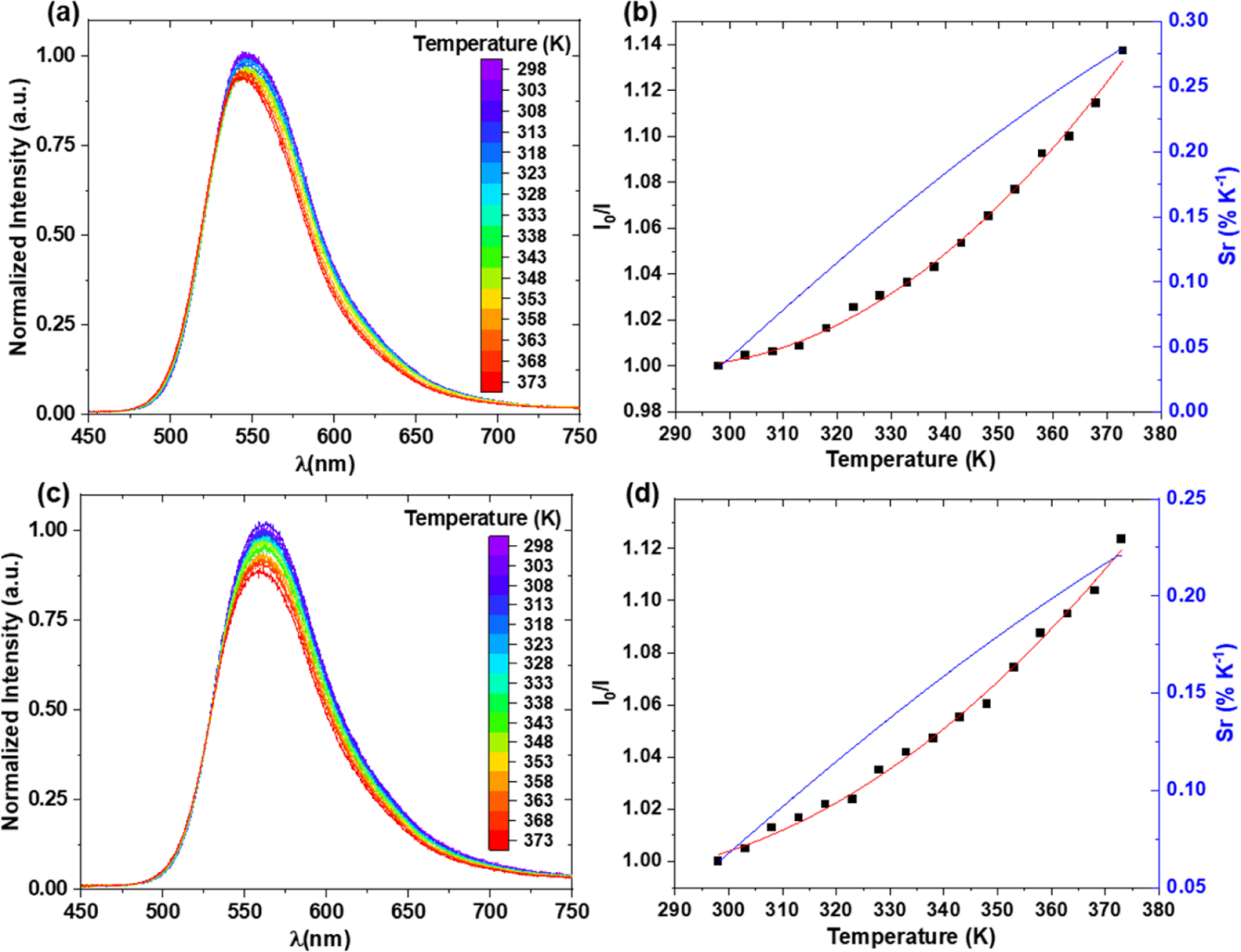
Emission spectra of 4Br-TPA-PZCN (a) and 4I-TPA-PZCN (c) recorded in the visible light range under 365 nm excitation (ambient pressure); LIR curves and the variation of relative sensitivity of 4Br-TPA-PZCN (b) and 4I-TPA-PZCN (d) derivatives with temperature.

A comparison of the fitted parameters reveals that both materials possess nearly identical linear terms, whereas 4Br-TPA-PZCN exhibits a slightly larger quadratic coefficient than 4I-TPA-PZCN, consistent with its marginally steeper LIR-T slope. Nevertheless, the overall relative sensitivities remain modest, with maximum *S*_r_ values of 0.28% K^–1^ for 4Br-TPA-PZCN and 0.22% K^–1^ for 4I-TPA-PZCN.

To further assess the temperature-sensing performance, both the absolute temperature uncertainty (δ*T*) and relative uncertainty were calculated using the same formulas as those applied for the pressure measurements. Figure 6a,b display the absolute and relative uncertainties, respectively. The minimum relative uncertainty is 0.79% K^–1^ for 4Br-TPA-PZCN and 0.62% K^–1^ for the 4I-TPA-PZCN, both occurring at 373 K, the upper end of the investigated temperature range. It indicates a high reliability of the LIR-T data and the associated fitting results. Under such low-uncertainty conditions, the observed LIR-T response remains modest, which suggests that the limited temperature sensitivity is an intrinsic characteristic of the materials rather than an artifact of experimental noise or data analysis. This further supports the conclusion that both materials exhibit weak temperature dependence.

**Figure 6:**
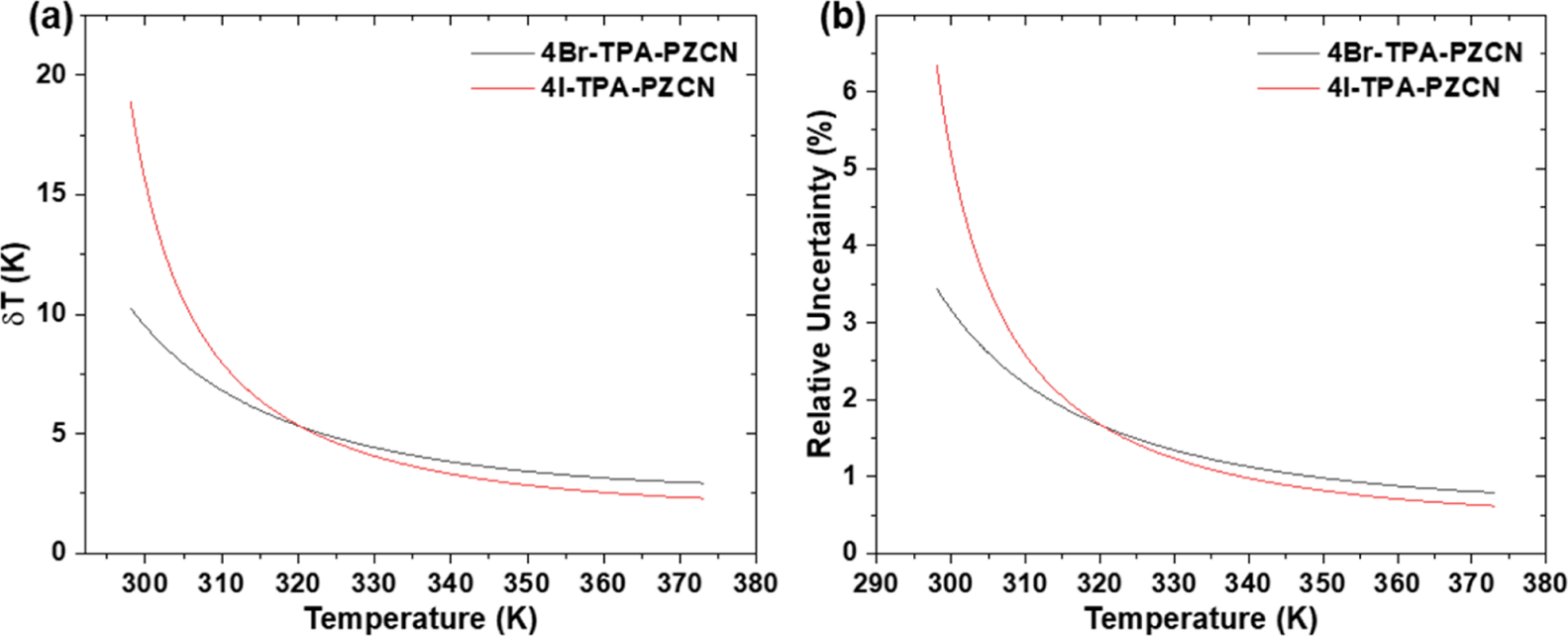
Absolute uncertainty - sensing resolution (a) and relative uncertainty in % (b) for the 4Br-TPA-PZCN and the 4I-TPA-PZCN within the operational temperature range.

Importantly, this weak temperature dependence directly translates into a key advantage for pressure sensing. Since the pressure readout parameter (LIR) exhibits only minor variations with temperature, the air-pressure sensing characteristics-including fitting parameters and sensitivity-remain effectively invariant over a broad temperature window. This intrinsic thermal stability minimizes temperature-induced cross-sensitivity, enabling reliable pressure measurements without additional temperature compensation. Therefore, while the thermal quenching behavior of 4Br-TPA-PZCN and 4I-TPA-PZCN is experimentally evident, its limited magnitude renders these materials more suitable as temperature-robust optical pressure sensors rather than high-sensitivity temperature probes.

## 4 Conclusions

In summary, this study investigated the pressure-dependent luminescence properties of two halogen-substituted TPA-PZCN derivatives, labeled 4Br-TPA-PZCN and 4I-TPA-PZCN. Thanks to the TADF phenomenon and the pronounced increase in emission under reduced oxygen conditions, we developed novel optical sensors for low pressure, operating from ca. 0.1 mbar to nearly 1 bar, i.e., over 4 orders of magnitude. The results show that halogen substitution modulates the energies and dynamics of the excited states, thereby significantly affecting the luminescent responses of the materials. By correlating the LIR with external pressure, the optical air-pressure sensing performance of both compounds was evaluated. 4Br-TPA-PZCN exhibits high sensitivity to pressure sensing, achieving a maximum *S*_r_ of up to 6.8% mbar^–1^ in the low-pressure range from 0.1 to 100 mbar, while 4I-TPA-PZCN reaches a maximum *S*_r_ of 1.8% mbar^–1^ over a narrower pressure range from 1 to 700 mbar. The minimum relative uncertainties were determined to be 7.86% at 6 mbar for 4Br-TPA-PZCN and 4.67% at 50 mbar for 4I-TPA-PZCN, confirming their effective operating ranges.

From a photophysical perspective, the pressure sensitivity of these TADF emitters arises from a well-balanced interplay between efficient triplet formation, slow yet competitive reverse intersystem crossing, and the diffusion-controlled quenching of triplet states by molecular oxygen in the ZNX matrix. Moreover, the distinct pressure sensitivities of the two emitters originate from differences in their triplet-state dynamics. The iodinated derivative generates approximately 1.5 times more triplet excitons than the brominated analogue and exhibits a slightly slower reverse intersystem crossing rate. As a result, a larger triplet population in 4I-TPA-PZCN resides longer in the excited state, increasing the probability of interaction with ^3^O_2_. This renders the iodinated emitter more sensitive to pressure variations at higher oxygen partial pressures, i.e., closer to atmospheric conditions. In contrast, the brominated derivative, characterized by faster triplet harvesting dynamics and a smaller triplet reservoir, responds more strongly at lower oxygen concentrations, leading to enhanced sensitivity in the low-pressure regime.

Temperature-dependent measurements indicate that thermal variations induce only weak changes in the luminescence response, rendering these materials unsuitable for standalone temperature sensing. Nevertheless, the limited influence of temperature ensures that reliable pressure measurements can be performed under different temperatures within the studied 298–373 K range. These findings indicate that halogen substitution provides an effective molecular design strategy to optimize the photophysical properties of organic phosphors, offering a solid basis for the development of high-performance optical pressure sensors with minimal temperature interference.

## Supplementary Material



## Notes

Areas under emission decay curves; fitting functions for pressure and temperature sensing; reaction scheme of 4Br-TPA-PZCN and 4I-TPA-PZCN; photoluminescence spectra, decay curves, and time-resolved emission spectra under different pressures; ^1^H and ^13^C NMR spectra of the synthesized compounds; The data supporting this article have been included (PDF)
