# Supplementary material for: Millisecond-Delayed Fluorescence in Heavy-Halogen-Substituted TADF Emitters for Air-Pressure Sensing
Source: ACS Appl Mater Interfaces. 2026 May 7;18(19):27866–76. doi: 10.1021/acsami.6c02198 (PMC13308879; doi:10.1021/acsami.6c02198)
Supplement: Supplementary file 1 [file am6c02198_si_001.pdf]

Supporting Information

Millisecond-Delayed Fluorescence in Heavy-Halogen-Substituted TADF Emitters for Air-Pressure Sensing

Fang Zhao[1], Christian Hernández-Álvarez[1], Illia E. Serdiuk[2,*], Michał Mońka,[2] Sebastian Mahlik[2], Marcin Runowski[1,*]

[1]*Adam Mickiewicz University, Faculty of Chemistry, Uniwersytetu Poznańskiego 8, 61-614 Poznań, Poland.*

[2]*Institute of Experimental Physics, Faculty of Mathematics, Physics and Informatics, University of Gdansk, Wita Stwosza 57, Gdansk, 80308 Poland.*

Corresponding author's email address

Marcin Runowski – Email: runowski@amu.edu.pl

Illia E. Serdiuk – Email: illia.serdiuk@ug.edu.pl

Table S1 Areas under emission decay curves

		p [bar]	Area Under Curve (S) PF	DF	Total	Total Vac/Air
**4Br-TPA-PZCN**	Air	1.01	5.41	0.22	5.63	
	Vac	10^{-9}	5.41	4.96	10.37	**1.84**
**4I-TPA-PZCN**	Air	1.01	3.62	0.21	3.83	
	Vac	10^{-9}	3.62	7.25	10.87	**2.83**

Table S2 The fitting functions for the pressure and temperature detection approach.

	Parameters	Fitting function	R^2
**4Br-TPA-PZCN**	Pressure	$LIR = 1.58 - 1.57/(1 - (p/9.89)^{0.93})$	0.99
**4I-TPA-PZCN**		$LIR = 2.23 - 1.23/(1 - (p/108.94)^{0.84})$	0.99
**4Br-TPA-PZCN**	Temperature	$LIR = 2.57 - 0.01T + 1.89\times10^{-5}T^2$	0.99
**4I-TPA-PZCN**		$LIR = 1.91 - 0.01T + 1.24\times10^{-5}T^2$	0.99

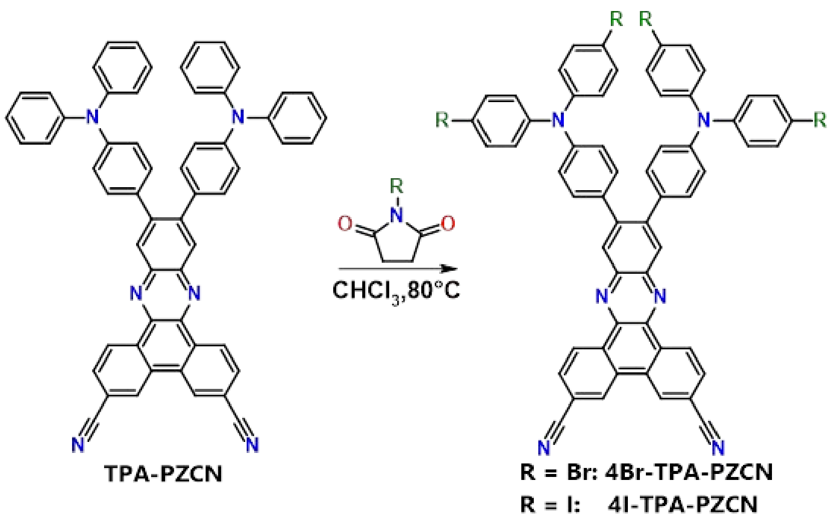

Figure S1 Schematic diagram of the reaction of 4Br-TPA-PZCN and 4I-TPA-PZCN

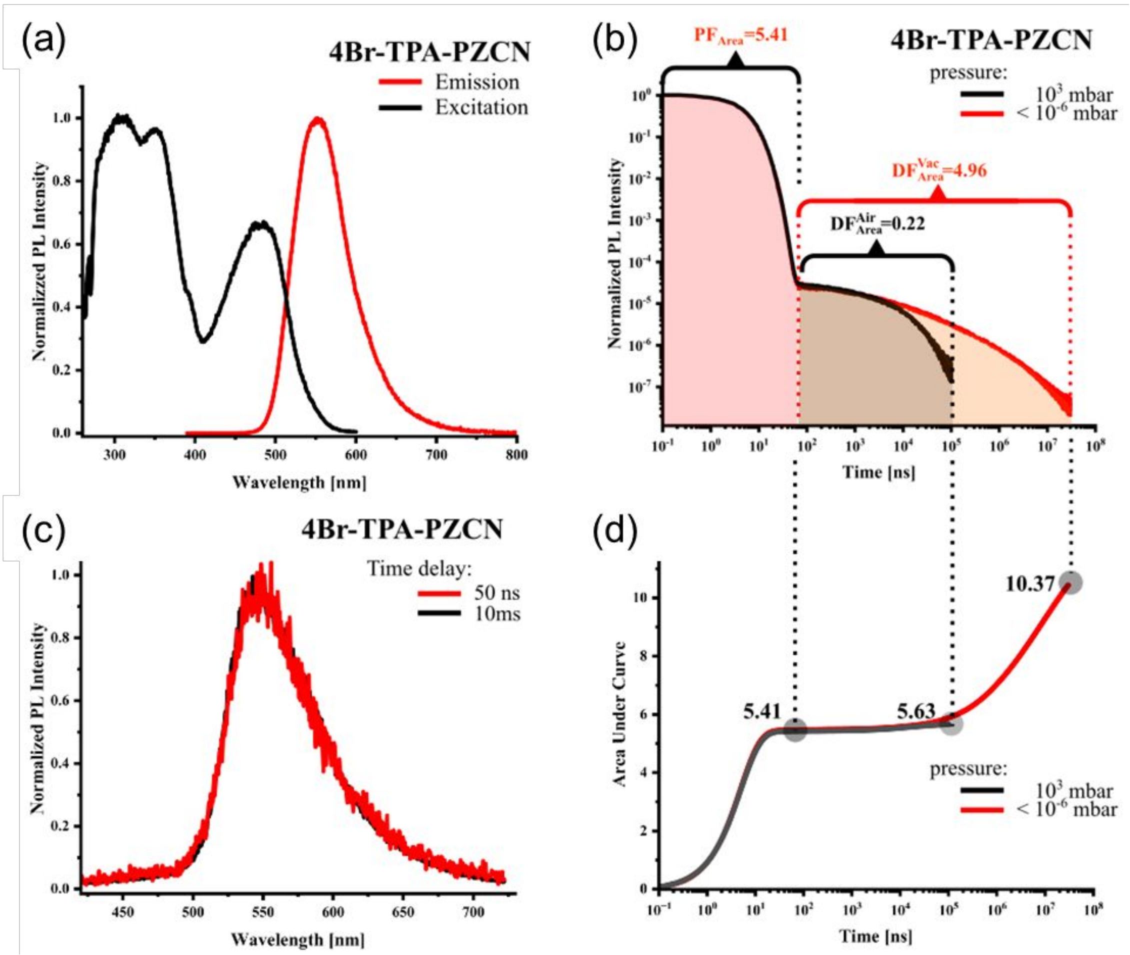

Figure S2 Photoluminescence features of **4Br-TPA-PZCN** in ZNX films (c = 0.1% *w/w*). (a) Steady-State excitation (λ_{obs} = 610 nm) and emission (λ_{exc} = 330 nm) spectra. (b) Photoluminescence intensity decays (λ_{exc} = 330 nm, 298 K) at normal and reduced pressure. (c) Time-resolved emission spectra (λ_{exc} = 330 nm, 298 K) recorded at different delay times. (d) Integrated emission intensities obtained as the area under the photoluminescence intensity decay curves of **4Br-TPA-PZCN** in air conditions and under vacuum.

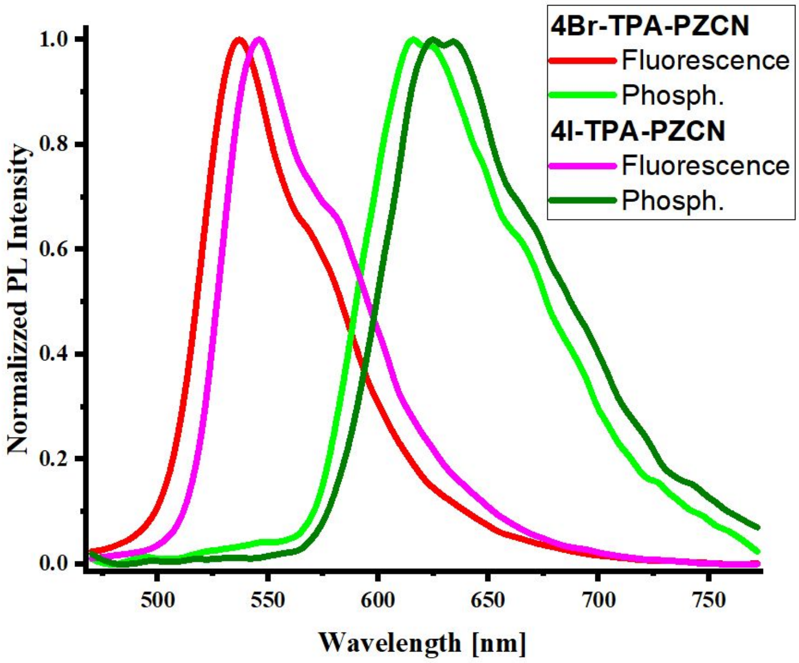

Figure S3 Photoluminescence spectra of emitters doped in ZNX films at 10 K: fluorescence is recorded within 1-50 ns after excitation, phosphorescence is recorded after 1 ms time delay.

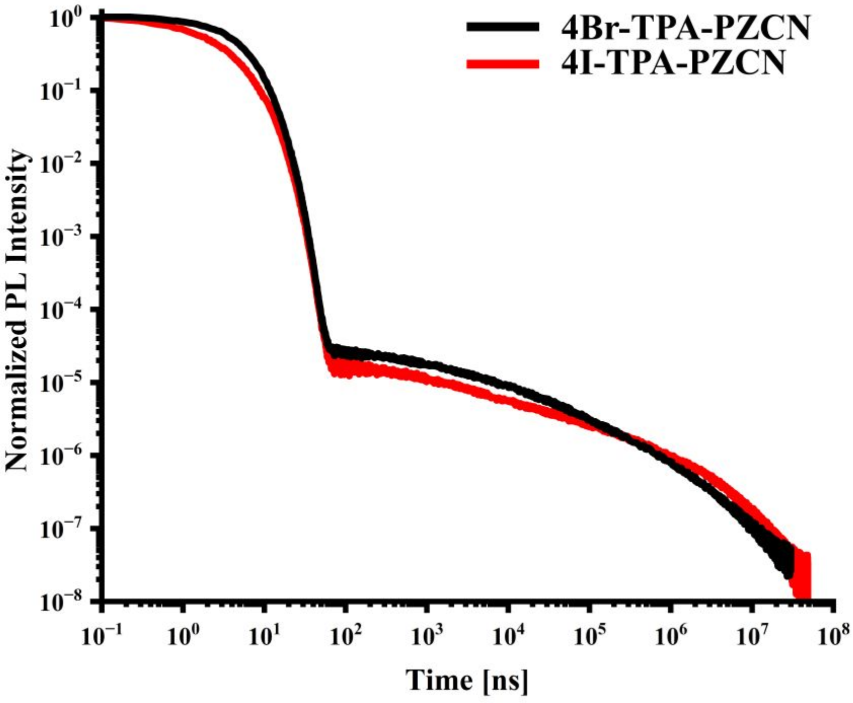

Figure S4 Photoluminescence intensity decays in ZNX under low pressure 10^{-6} mbar, 298K, λ_{exc} = 330 nm.

NMR spectra

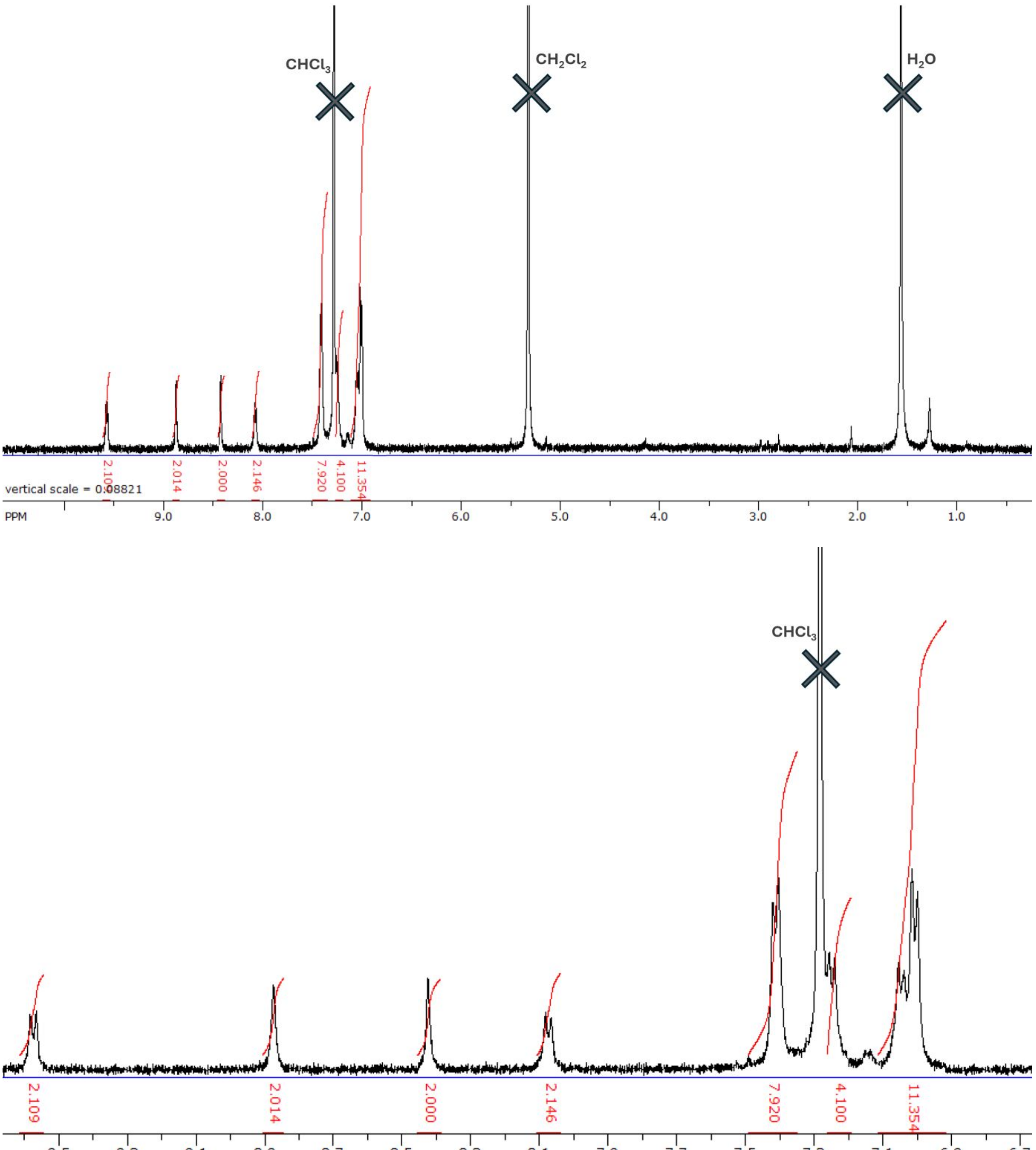

Figure S5 ^1H NMR spectrum of 4Br-TPA-PZCN in $CDCl_3$

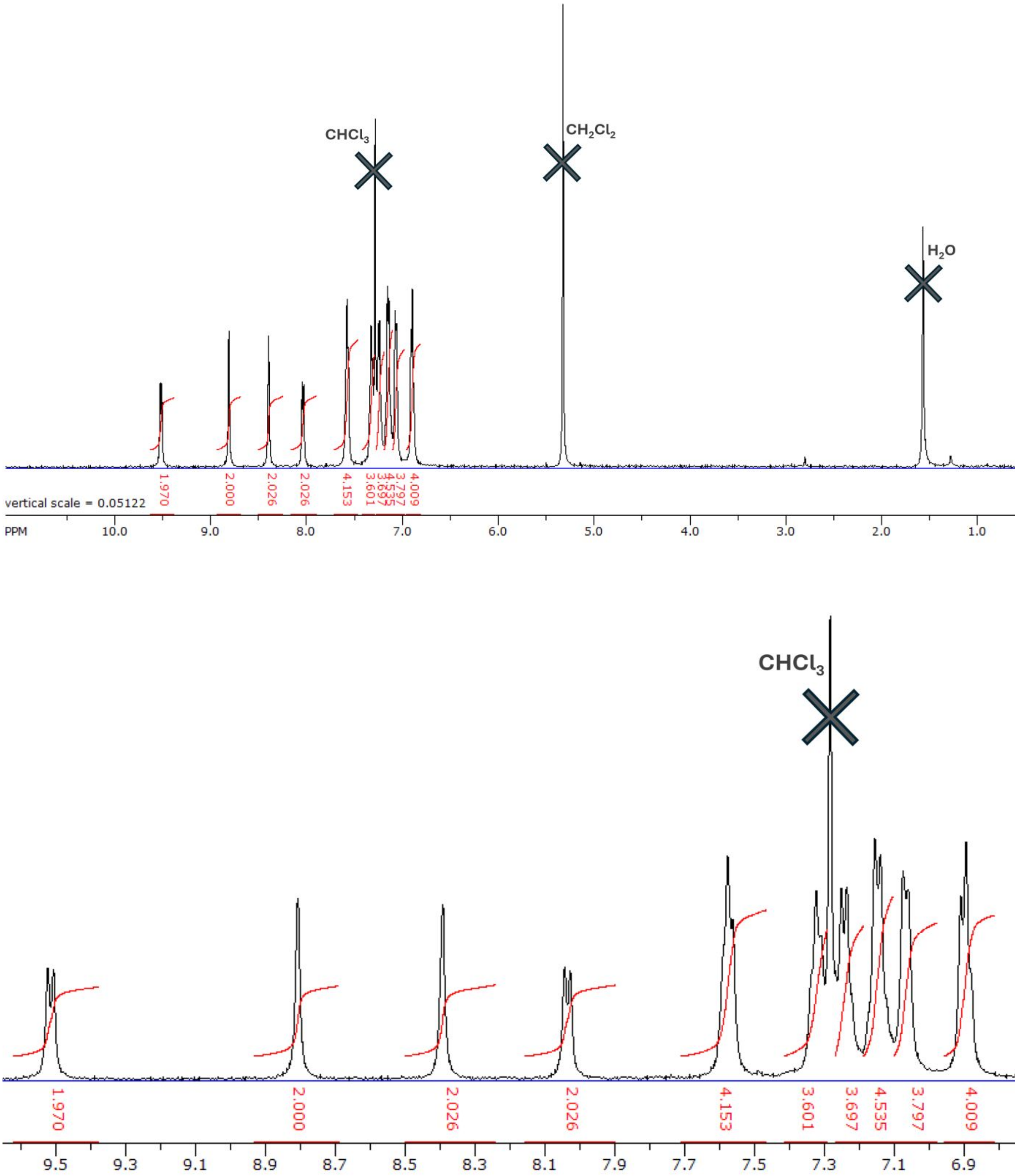

Figure S6 ^{1}H NMR spectrum of 4I-TPA-PZCN in $CDCl_3$

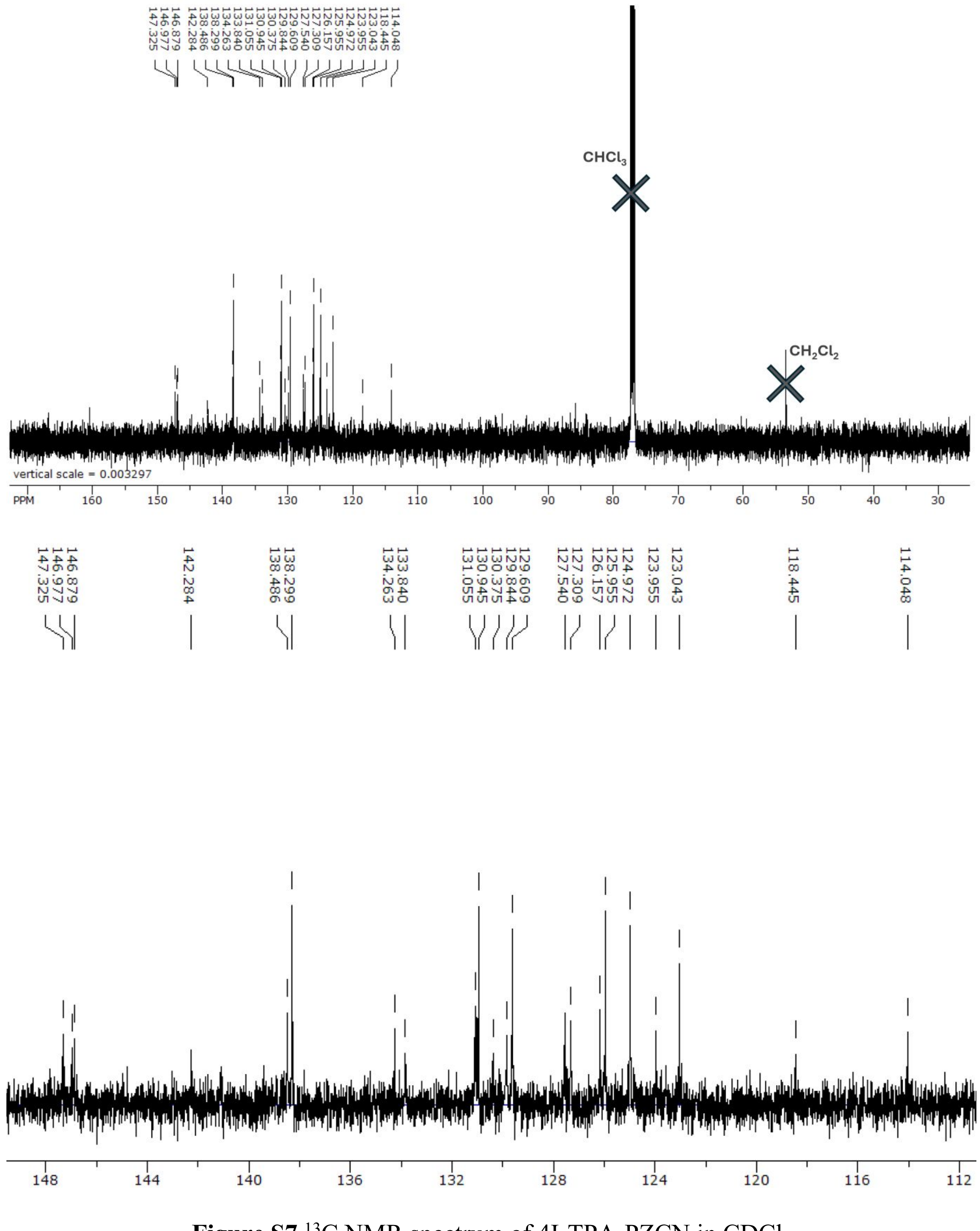

Figure S7 ^{13}C NMR spectrum of 4I-TPA-PZCN in $CDCl_3$

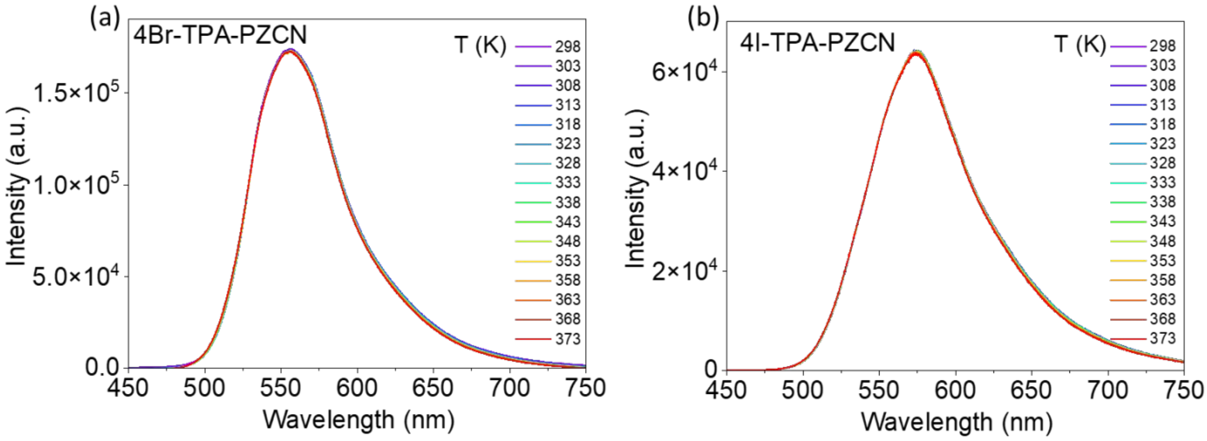

Figure S8 Temperature-dependent photoluminescence spectra of (a) 4Br-TPA-PZCN and (b) 4I-TPA-PZCN under vacuum conditions (≈10^{-6} bar).

Determination of photophysical parameters

PL dintesity decay curves showed were fitted with the multiexponential equation:

$$I(t) = A_0 + \sum_{i=1}^{n} A_i \exp\left(-{}^{t}/_{\tau_i}\right), \tag{S1}$$

where A_i is the pre-exponential factor, τ_i is the decay time and $I(t)$ is emission intensity. Average lifetimes of prompt (τ_{PF}) and delayed fluorescence (τ_{DF}) were determined using the following formula:

$$\tau_{PF,\,DF} = \sum_{i=1}^{n} f_i \tau_i\,, \tag{S2}$$

where $f_i$is fractional contribution of i-th component expressed as:

$$f_i = \frac{A_i \tau_i}{\sum_{i=1}^{n} A_i \tau_i}. \tag{S3}$$

The ratio of DF and PF quantum yields $\varphi_{DF}/\varphi_{PF}$ was determined as follows:

$$\frac{\varphi_{DF}}{\varphi_{PF}} = \frac{A_{DF}\tau_{DF}}{A_{PF}\tau_{PF}}, \tag{S4}$$

where A_{DF} and A_{PF} are pre-exponential factors of delayed and prompt fluorescence, respectively. Rate constants of radiative (k_r) and nonradiative (k_{nr}) decay and intersystem crossing (k_{ISC}) are given by equations[1]

$$k_r = \frac{\varphi_{PF}}{\tau_{PF}}, \tag{S5}$$

$$k_{ISC} = \frac{\varphi_{DF}}{\varphi\tau_{PF}}, \quad \text{(S6)}$$

$$k_{nr} = \frac{1}{\tau_{PF}} - (k_r + k_{ISC}), \quad \text{(S7)}$$

where φ is PLQY ($\varphi_{DF} + \varphi_{PF}$). Further, the quantum yields for ISC and rISC are calculated as:

$$\varphi_{ISC} = k_{ISC}\tau_{PF}, \quad \text{(S8)}$$

$$\varphi_{rISC} = \frac{1 - \varphi_{PF}/\varphi}{\varphi_{ISC}}. \quad \text{(S9)}$$

Finally, rISC rate constant (k_{rISC}) can be calculated as:

$$k_{rISC} = \frac{\varphi_{rISC}}{\tau_{DF}}\left(\frac{\varphi}{\varphi_{PF}}\right). \quad \text{(S10)}$$

SI References

[1] Y. Tao, K. Yuan, T. Chen, P. Xu, H. Li, R. Chen, C. Zheng, L. Zhang, W. Huang, *Adv. Mater.* **2014**, *26*, 7931.
